# Supplementary material for: Impact of surgical site infection (SSI) following gynaecological cancer surgery in the UK: a trainee-led multicentre audit and service evaluation
Source: BMJ Open. 2019 Jan 24;9(1):e024853. doi: 10.1136/bmjopen-2018-024853 (PMC6347877; doi:10.1136/bmjopen-2018-024853)
Supplement: Supplementary file 1 [file bmjopen-2018-024853supp001.pdf]

Supplementary Table A: Modified surgical complexity scoring system based upon complexity and number of surgical procedures performed

Adapted from [6]

Procedure	Points
Total Hysterectomy- Bilateral Salpingo-Oophorectomy (TH-BSO)	1
Omentectomy	1
Pelvic lymphadenectomy	1
Paraaortic lymphadenectomy	1
Pelvic peritoneum stripping	1
Abdominal peritoneum stripping	1
Rectosigmoidectomy EEA	3
Large bowel resection	2
Diaphragm stripping/resection	2
Splenectomy	2
Liver resection	2
Small bowel resection	1
Resection of tail of pancreas	5
Cholecystectomy	5
Partial gastrectomy	5

Complexity score groups

1. Standard/ (low) ≤3

2. Intermediate 4-8

3. Ultra-radical (high) ≥8

Supplementary Table B:

(adapted from [7])

Term	Definition	Criteria (Any 1 of criteria below)
Superficial incisional infection	SSI within 30 days of surgery involving the skin or subcutaneous tissue of the incision	1. Purulent drainage from incision<br>2. Positive microbiological culture/pus cells present on swab from incision<br>3. Two of:<br>a. pain or tenderness<br>b. swelling<br>c. redness<br>4. the superficial incision is deliberately opened by a surgeon to manage the infection<br>5. clinician diagnoses a superficial incisional infection.
Deep incisional infection:	SSI involving the deep tissues (i.e. fascial and muscle layers) that occurs within 30 days of surgery<br>.	1. Purulent drainage from the deep incision<br>2. Positive microbiological culture/pus cells present on swab from fluid from deep incision<br>3. Spontaneous dehiscence/opening of wound by surgeon AND patient has at least 1 of:<br>a. fever (>38°C)<br>b. localized pain or tenderness<br>4. Radiologically, histologically or surgically diagnosed abscess involving the deep incision.<br>5. Clinician diagnoses a deep incisional SSI
Deep organ space infection	SSI involving any part of the body deeper than the fascial and muscle layers that was opened or manipulated during the operative procedure occurring within 30 days of the procedure	Patient has at least two of the following: fever (>38.0°C), hypotension, nausea, vomiting, abdominal pain or tenderness AND has either organisms identified from tissue or fluid or positive blood cultures and radiological evidence of abscess or infection
Patient reported SSI	SSI reported by patient to have occurred within 30 days of surgery	1. Discharge pus AND antibiotics prescribed<br>2. Clinical signs* AND dehiscence<br>3. Clinical signs* AND antibiotics prescribed<br><br>*clinical signs – at least 2 of the following must be present: pain, heat, redness or swelling
