# Supplementary material for: Impact of surgical site infection (SSI) following gynaecological cancer surgery in the UK: a trainee-led multicentre audit and service evaluation
Source: BMJ Open. 2019 Jan 24;9(1):e024853. doi: 10.1136/bmjopen-2018-024853 (PMC6347877; doi:10.1136/bmjopen-2018-024853)
Supplement: Author's manuscript [file bmjopen-2018-024853.draft_revisions.pdf]

BMJ Open is committed to open peer review. As part of this commitment we make the peer review history of every article we publish publicly available.

When an article is published we post the peer reviewers' comments and the authors' responses online. We also post the versions of the paper that were used during peer review. These are the versions that the peer review comments apply to.

The versions of the paper that follow are the versions that were submitted during the peer review process. They are not the versions of record or the final published versions. They should not be cited or distributed as the published version of this manuscript.

BMJ Open is an open access journal and the full, final, typeset and author-corrected version of record of the manuscript is available on our site with no access controls, subscription charges or pay-per-view fees (http://bmjopen.bmj.com).

If you have any questions on BMJ Open's open peer review process please email editorial.bmjopen@bmj.com

BMJ Open

IMPACT OF SURGICAL SITE INFECTION (SSI) FOLLOWING GYNAECOLOGICAL CANCER SURGERY IN THE UNITED KINGDOM: A TRAINEE-LED MULTICENTRE AUDIT AND SERVICE EVALUATION

Journal:	*BMJ Open*
Manuscript ID	bmjopen-2018-024853
Article Type:	Research
Date Submitted by the Author:	17-Jun-2018
Complete List of Authors:	O'Donnell, Rachel; Northern Gynaecological Oncology Centre; Surgical Gynaecological Oncology Research Network<br>Angelopoulos, Georgios; Surgical Gynaecological Oncology Research Network; James Cook University Hospital<br>Beirne, James; Surgical Gynaecological Oncology Research Network; Northern Ireland Centre for Gynaecological Oncology, Belfast City Hospital<br>Biliatis, Ioannis; Surgical Gynaecological Oncology Research Network; Royal Marsden NHS Foundation Trust,<br>Bolton, Helen; Surgical Gynaecological Oncology Research Network; Addenbrooke's Hospital<br>Bradbury, Melissa; Surgical Gynaecological Oncology Research Network; Northern Gynaecological Oncology Centre<br>Craig, Elaine; Surgical Gynaecological Oncology Research Network; Northern Ireland Centre for Gynaecological Oncology, Belfast City Hospital<br>Gajjar, Ketan; Surgical Gynaecological Oncology Research Network; Addenbrooke's Hospital<br>Mackintosh, Michelle; Surgical Gynaecological Oncology Research Network, ; St Mary's Hospital, Gynaecological Oncology<br>MacNab, Wendy; Surgical Gynaecological Oncology Research Network; Glasgow Royal Infirmary<br>Madhuri, Thumuluru Kavitha; Surgical Gynaecological Research Network; Royal Surrey County Hospital NHS Foundation Trust, Department of Gynaecological Oncology<br>McComiskey, Mark; Surgical Gynaecological Oncology Research Network; St Mary's Hospital, Gynaecological Oncology<br>Myriokefalitaki, Eva; Surgical Gynaecological Oncology Research Network; University Hospitals of Leicester NHS Trust, Gynaecological Oncology<br>Newton, Claire; Surgical Gynaecological Oncology Research Network; Saint Bartholomew's Hospital, Gynaecological Oncology<br>Ratnavelu, Nithya; Surgical Gynaecological Oncology Research Network; Northern Gynaecological Oncology Centre<br>Taylor, Sian; Surgical Gynaecological Oncology Research Network; Liverpool Womens NHS Foundation Trust, Gynaecological Oncology<br>Thangavelu, Amudha; Surgical Gynaecological Oncology Research Network; Nottingham University Hospitals NHS Trust, Gynaecological Oncology<br>Rhodes, Sarah; Centre for Biostatistics, Faculty of Biology, Medicine and Health<br>Crosbie, Emma; Surgical Gynaecological Oncology Research Collaboration;

University of Manchester Faculty of Biology, Medicine and Health, Gynaecological Oncology
Edmondson, Richard; Surgical Gynaecological Oncology Research Network; University of Manchester Faculty of Biology, Medicine and Health, Gynaecological Oncology
Wan, Yee-Loi; University of Manchester Faculty of Biology, Medicine and Health, Gynaecological Oncology

Keywords: WOUND MANAGEMENT, SURGERY, Gynaecological oncology < GYNAECOLOGY, surgical site infection, noscomial infection, hospital acquired infection

SCHOLARONE™
Manuscripts

For peer review only

IMPACT OF SURGICAL SITE INFECTION (SSI) FOLLOWING GYNAECOLOGICAL CANCER SURGERY IN THE UNITED KINGDOM: A TRAINEE-LED MULTICENTRE AUDIT AND SERVICE EVALUATION

Authors

RL O'Donnell 1,2, G Angelopoulos 1,3, JP Beirne 1,4, I Biliatis 1,5, H Bolton 1,6, M Bradbury 1,2, E Craig 1,4, K Gajir 1,6, ML MacKintosh 1,7, W MacNab 1,8, K Madhuri 1,9, M McComiskey 1,7, E Myriokefalitaki 1,10, C Newton 1,11, N Ratnavelu 1,2, S Taylor 1,12, A Thangavelu 1,13, SA Rhodes 14, EJ Crosbie 1,15, RJ Edmondson 1,15, YL Wan 1,15

1. Surgical Gynaecological Oncology Research Network
2. Northern Gynaecological Oncology Centre, Gateshead Foundation NHS Trust, Gateshead NE9 6SX
3. James Cook Hospital, Marton Road, Middlesbrough, TS4 3BW
4. Northern Ireland Centre for Gynaecological Cancer, Belfast City Hospital, Lisburn Road, Belfast, BT9 7AB
5. Royal Marsden Hospital, Fulham Road, London, SW3 6JJ
6. Addenbrooke's Hospital, Hills Road, Cambridge, CB2 0QQ
7. St Mary's Hospital, Manchester University Hospitals NHS Foundation Trust, Manchester Academic Health Science Centre; Oxford Road, Manchester, M13 9WL
8. Glasgow Royal Infirmary, Castle Street, Glasgow, G4 0SF
9. Royal Surrey County Hospital, Egerton Rd, Guildford GU2 7XX
10. University Hospital Leicester, Infirmary Square, Leicester LE1 5WW
11. St Bartholomew's Hospital, West Smithfield, London EC1A 7BE
12. Liverpool Women's Hospital, Crown St, Liverpool L8 7SS
13. Nottingham University Hospital, Hucknall Rd, Nottingham NG5 1PB
14. Centre for Biostatistics, Division of Population Health, Health Services Research and Primary Care, University of Manchester, Oxford Road, Manchester M13 9PL
15. Division of Cancer Sciences, Faculty of Biology, Medicine and Health, University of Manchester, M13 9WL

Corresponding author: Y Louise Wan

Corresponding author email address: louise.wan@manchester.ac.uk

For peer review only - http://bmjopen.bmj.com/site/about/guidelines.xhtml

For peer review only

Abstract

Objectives

Surgical site infection (SSI) complicates 5% of all surgical procedures in the United Kingdom (UK), and is not only a major cause of post-operative morbidity, but also a substantial drain on healthcare resources. Little is known about the incidence of SSI and its consequences in women undergoing surgery for gynaecological cancer. Our aim was to perform the first national audit of SSI following gynaecological cancer surgery through the establishment of a UK-wide trainee-led research network.

Design and setting

In a prospective audit, we collected data from all women undergoing laparotomy for suspected gynaecological cancer at 12 specialist oncology centres in the UK during an eight-week period in 2015. Clinicopathological data were collected and wound complications and their sequelae were recorded during the 30 days following surgery.

Results

In total, 339 women underwent laparotomy for suspected gynaecological cancer during the study period. A clinical diagnosis of SSI was made in 54 (16%) women. 92.6% (50/54) of women with SSI had prolonged hospital stays and 6/23 (26.1%) had their adjuvant treatment delayed or cancelled. Multivariate analysis found BMI was the strongest risk factor for SSI [OR 1.08 (95% CI 1.03 to 1.14) per 1kg/m^2 increase in BMI, (p=0.001)]. Wound drains [OR 2.92(95% CI 1.41 to 6.04), p=0.004] and staple closure [OR 3.13 (95% CI 1.50 to 6.56), p=0.002] were also associated with increased risk of SSI.

Conclusions

SSI is common in women undergoing surgery for gynaecological cancer leading to delays in discharge and adjuvant treatment. Resultant delays in adjuvant treatment may impact cancer-specific survival rates. Modifiable factors, such as choice of wound closure material offer opportunities for reducing SSI and reducing morbidity in these women. There is a clear need for new trials in SSI prevention in this patient group; our trainee-led initiative provides a platform for their successful completion.

Abstract word count: 299

Strengths & Limitations of this study

- This study documents real-world clinical practice in the prevention and management of surgical site infection (SSI) in women undergoing open gynaecological cancer surgery in the UK.

For peer review only - http://bmjopen.bmj.com/site/about/guidelines.xhtml

- Data were collected prospectively and consecutively without exclusions according to comorbidities or treatment from all women undergoing gynaecological cancer surgery at 12 UK centres, encompassing the various national care settings (except Wales).
- Data relating to direct and indirect health care costs of SSIs were not available.
- Further longitudinal data examining the cancer specific outcomes of this cohort are not currently available.

Funding Statement

YLW was funded by the Wellcome Trust as part of a Wellcome Trust Clinical Research Fellowship (101714/Z/13/Z). Patient involvement was funded by a NIHR North West Research Design Service Patient and Public Involvement Bursary. Neither funder was involved in the planning or execution of the study or the drafting of the manuscript.

Competing interests

No competing interests.

Contributions

ROD, YLW, MB, KM, AT, MLM, MM conceived and designed the study. Data was collected by ROD, NR, GA, MB, IB, HB, JB, EC, KG, WM, KM, MLM, MM, EM, CN, NR, ST and AT and analysed by ROD, MM, KM, SR and YLW. The article was drafted by ROD and YLW and revised and approved by all authors.

Data sharing

Original data are available from the corresponding author on request.

Article word count

2854

For peer review only

For peer review only - http://bmjopen.bmj.com/site/about/guidelines.xhtml

Introduction

Gynaecological cancers collectively account for 11% of all cancers affecting women in the UK. Surgery remains the mainstay of treatment and despite recent advances in surgical technologies, with an increase in the use of minimally invasive techniques, a large proportion of patients still require laparotomy. For many women, their treatment will involve a hysterectomy with an associated high risk of surgical site infection (SSI) as a result of the inherently contaminated nature of procedures involving entry into the genital tract.

Although most women undergoing laparotomy can expect their wound to heal without any problem, there is a lack of high quality data in gynaecological oncology to indicate the incidence and consequences of SSI. A study by Iyer et al, which aimed to identify predictors of complications in women undergoing surgery for gynaecological malignancy, describes Grade II-IV infections in 27.8% of all gynaecological surgery cases accounting for 31% of all complications [1]. Although this study identified predictors of complications, the consequences of such complications were not assessed. The need to identify preventable complications coupled with effective interventions remains. The importance of SSI prevention, across all surgical specialities, has also been acknowledged by the UK government with the publication of various national guidelines outlining care bundles with recommended interventions during the pre-operative, intraoperative and postoperative phases of the patient journey[2,3]. This has led to a downward trend in the incidence of SSIs in most types of surgery [4]. However, worryingly, there has been a year on year rise in SSI related to abdominal hysterectomies since 2014 [4].

Further research into understanding the direct and indirect effects of SSI upon the gynaecological oncology population and the additional interventions that could reduce surgical site infections is, therefore, urgently needed. An innovative UK-wide multi-centre collaborative of trainees and consultants with an interest in clinical research in gynaecological oncology was established in 2015 to develop and implement evidence based interventions to improve surgical care for women undergoing gynaecological cancer surgery.

The primary objective of this study was to define the incidence of SSI in the UK gynaecological oncology population undergoing laparotomy and record standard practices. The secondary objectives were to identify risk factors associated with infection and assess the potential impact upon length of stay and delay to adjuvant cancer treatment. This study also aimed to provide baseline data to develop a modified enhanced recovery programme for women undergoing gynaecological cancer surgery.

Methods

This prospective multi-centre audit of all consecutive women undergoing laparotomy for suspected or confirmed gynaecological malignancy was undertaken across 12 of the 44 UK tertiary centres during an 8-week period September - November 2015. Daily review of surgical cases was undertaken in each institute and cross referenced with theatre IT systems to ensure all subjects were included. Advice was sought from local ethics committees, who confirmed that this study was exempt from ethical review as the research was limited to the

For peer review only - http://bmjopen.bmj.com/site/about/guidelines.xhtml

secondary use of fully anonymised information previously collected in the course of normal care without the intention to use it for research at the time of collection.

Local audit leads were recruited via the British Gynaecological Cancer Society Trainees group and the Tomorrows Leaders in Surgical Research workshops. These local leads co-ordinated the prospective collection and coding of anonymised data using a standardised data-collection tool developed in line with NICE guidance [2]. Additional information including patient demographics, comorbidities, surgicopathological and wound complication data were also contemporaneously collected. All patients were categorised using the derived patient status risk index described by the American Society of Anaesthesiologists (ASA) [5] and their wound classified as per the CDC wound classification system (Supplementary Table A) [6]. Surgical complexity was classified using a modification of the surgical scoring system (Supplementary Table B) developed by the Mayo clinic [7] for ovarian cancer. Each individual procedure was given a score and the total score for the surgery was the sum of the individual scores. Surgeries were categorised into three groups of increasing complexity based on the overall score (Low - score ≤3; Intermediate – score 4-8; Radical - ≥8). In addition to the procedure, the duration of surgery and estimated blood loss (EBL) were also recorded.

The Centres for Disease Control and Prevention (CDC) definitions for superficial incisional, deep incisional and organ/space SSI were used to standardise diagnosis of SSI across all centres[8,9]. A modified definition was applied for patient reported SSI, as per the Public Health England Surgical Site Infection Surveillance programme protocol [9]. The impact of SSI on length of stay, return to theatre, use of negative pressure wound therapy, admission to intensive care and time to adjuvant chemo or radiotherapy was studied.

To ensure completeness of data and to capture events following discharge, all recorded interactions (i.e. telephone consultations/clinic visits/hospital records) with patients in the 30 days post-operatively were analysed. Data from patient reported infection following discharge as well as observed infection during admission were included in the analysis.

Statistical analysis

Descriptive analysis of clinico-pathological data was undertaken to characterise the cohort and the predictive value of each risk factor for SSI was separately assessed using univariable analysis by logistical regression or Chi^2 as appropriate. The variables to include in the logistic regression were based on the univariate analysis and known risk factors for SSI. To take into account variations in the length of time to last recorded contact, the incidence density was calculated using the number of cases in the numerator and the total number of days of patient follow-up (from inpatient surveillance) in the denominator giving the number of SSIs per 10 patient days of follow-up.

Patient involvement

No patients were directly involved in this research. However, the views of focus groups were sought regarding the most appropriate sequelae of SSI to measure. Patients are actively involved in the design of potential interventions to prevent SSIs and any future trials from our trainee-led initiative.

For peer review only - http://bmjopen.bmj.com/site/about/guidelines.xhtml

Results

Data Collection

During the prospective study, undertaken between 1st September 2015 and 1st November 2015, with follow-up until 1st December 2015, 12 UK tertiary centres submitted data for a total of 339 patients. Data were complete in 98.0% of fields. Median time until last patient encounter was 22 days (Interquartile range (IQR) 14-41 days). The final patient contact in the post-operative follow up period for most patients was at their outpatient review 4-6 weeks after surgery.

Participants

Baseline patient characteristics are shown in Table 1. The median patient age was 62 years (IQR 53–71) with a median body mass index (BMI) of 28.0 kg/m^2 (IQR 24.0 – 32.8). Pre-operative performance status varied with 62 (18.3%) patients classified as ASA 1; 166 (49.0%) as ASA 2; and 87 (25.7%) as ASA 3. 13% of patients were hypoalbuminaemic (albumin <35g/dL).

The majority of surgeries were undertaken as primary treatments (244, 72%) with 69 (20.4%) patients undergoing interval surgery after neoadjuvant chemotherapy for ovarian cancer. 22 (6.5%) patients underwent surgery for recurrent disease with palliative intent (from all primary sites). Of the 339 study patients, 288 (85.0%) had a diagnosis of cancer confirmed, the majority of whom, 200 (59%), had a diagnosis of ovarian cancer, Table 1.

For peer review only

For peer review only - http://bmjopen.bmj.com/site/about/guidelines.xhtml

Table 1: Patient Demographics

Characteristic	Cases (%) – unless otherwise stated
**Age**	n=339
Age (years), median (interquartile range)	62 (51-71)
**BMI**	n=323
BMI (kg/m^2), median (interquartile range)	28.0 (15.0 – 60.6)
ASA	n=315
1	62 (18.3)
2	166 (49.0)
3	87 (25.7)
Co-morbidity	n=334
Diabetes	34 (10.0)
Oral hypoglycaemic / insulin use	21 (6.2) / 13 (3.8)
Thyroid dysfunction	43 (12.7)
Immune defect	2 (0.6)
Pre-operative anaemia	34 (10.0)
Coagulopathy	9 (2.7)
Chronic kidney disease	15 (4.4)
Chronic lung disease	37 (10.9)
Current smoker	36 (10.6)
Other non-active malignancy	16 (4.7)
Diagnosis	n=337
Ovarian /Primary peritoneal carcinoma	200 (59.3)
Endometrial cancer	61 (18.1)
Cervical	9 (2.7)
Benign	51 (15.1)
Other non-gynaecological cancer	18 (5.3)
FIGO Stage	n=325
1	83 (25.5)
2	23 (7.1)
3	129 (39.7)
4	24 (7.4)
Not applicable	66 (20.3)
Surgical radicality	n=338
Standard (≤3)	265 (78.4)
Intermediate (4 - 8)	61 (18.0)
Ultra-radical (≥8)	13 (3.8)
**Procedure**	n=338
Hysterectomy	272 (80.2)
Oophorectomy/salpingectomy	305 (90.0)
Omentectomy	250 (73.7)
Pelvic lymph node dissection	67 (19.8)
Para-aortic lymph node dissection	35 (10.3)
Small bowel resection	19 (5.6)
Large bowel resection	36 (10.6)
Peritoneal stripping	55 (16.2)
Panniculectomy	3 (0.9)
Colostomy	15 (4.4)
Urostomy	2 (0.6)

Abbreviations. ASA: American Society of Anaesthesiologists; BMI: body mass index.

For peer review only

For peer review only - http://bmjopen.bmj.com/site/about/guidelines.xhtml

Infection prophylaxis

All patients were screened for MRSA pre-operatively, in compliance with NICE guidance, and colonisation with MRSA was confirmed in 2 patients who subsequently underwent pre-operative eradication. Infection prophylaxis policy varied between centres with variations in surgical skin preparation used and in the use of prophylactic antibiotics. Povidine iodine or chorhexidine were used in 113 (33.3%) and 212 (62.5%) of cases, respectively. 318/339 (93.8%) patients received intravenous antibiotics at induction of anaesthesia, of which 292/318 (91.8%) was in line with local microbiology policy. Of those who received prophylactic intra operative antibiotics, 23 (7.2%) received triple therapy, 85 (26.7%) received combined double therapy and 210 (66.0%) received single agent prophylaxis. The most frequently used antibiotic was co-amoxiclav, used alone or in combination in 236 (69.6%) patients. 306 (90.3%) surgeries were classified as clean-contaminated or contaminated.

Surgery

The majority of laparotomy incisions were midline and extended above the level of the umbilicus to the pubis. Median operating time was 150 minutes (IQR 110-191 minutes). The median surgical complexity score for the entire cohort was 2 (Range 1–17, IQR 2-3), Table 1. 74 (21.8%) patients had a radicality score of ≥4 which included 50 (15.6%) patients who underwent bowel resection, Table 1.

Post-operative wound management

Use of wound drains and skin closure techniques was highly variable and the incidence of drain use, skin closure and dressing used are shown in Table 2. Dressing choice was largely dependent upon NHS site, with little variation within each institution. The majority of dressings were occlusive and removed on the second post-operative day. However, 7 patients had surgical glue preparations, with or without an occlusive dressing. Additionally, 7 patients, from 3 different institutions used a PICO negative pressure wound dressing (Smith and Nephew).

Table 2: Post-operative wound management

Technique		Usage n (%)		Missing data (n)
**Intra-abdominal drain**		59	17.4	7
**Subcutaneous tissue closure**		68	20.1	7
**Local anaesthetic wound catheter**		16	4.7	7
**Skin closure**	*Staples*	174	51.3	0
	Subcuticular monocryl	164	48.4	
	Other	1	0.3	
**Wound dressing**	*Mepore*	145	42.8	2
	Tegaderm	68	20.1	
	Opsite	59	17.4	
	Other	63	18.6	

For peer review only - http://bmjopen.bmj.com/site/about/guidelines.xhtml

Wound infections

Of the 339 patients included in the study, 54 (15.9%) were diagnosed with a surgical site infection by CDC criteria and 30 (8.8%) had wound dehiscence. 20 (5.9%) patients had dehiscence in the presence of infection. To take into account the variation in the length of follow up, incidence density was calculated; 0.5 cases of infection per 10 days of patient follow-up was seen. Severity of infection was variable with the incidence of various markers of severity shown in Table 3. Infection was most prevalent in patients undergoing laparotomy for endometrial cancer, with an incidence of 21.3% in this subgroup. However, there was no significant difference in incidence of SSI by site of tumour origin, p=0.7358.

Causative micro-organisms were isolated from wound culture in only 32/54 cases (59.3%). The most prevalent culture result showing mixed growth predominantly of Escherichia Coli and/or other bowel commensals. Staphylococcus aureus was a reported cause of SSI in 4 cases in this series.

Table 3: Markers of SSI severity

Infection type and Severity n=54			**Cases (%) -unless otherwise stated**
**Infection**	Cultured organisms	*Positive culture*	32 (59.3)
	Time of infection	*Post-op admission*	24 (44.4.)
		Community	25 (46.3)
		Local hospital readmission	4 (7.4)
		Central hospital readmission	1 (1.9)
	Antibiotic route	*IV*	24 (44.4)
		PO	25 (46.3)
		None	6 (11.1)
	Length of course	*Days, median (range)*	7 (5 – 14)
**Severity**	Pyrexia	*Temp >38°C*	11 (20.4)
	WBC	*x10/L, median (interquartile range)*	10 (7 – 14)
	Cellulitis	*Clinical diagnosis*	24 (44.4)
	Skin dehiscence	*Clinical diagnosis*	30 (55.6)
		Treated with packing	14 (25.9)
		Managed without packing	16 (29.6)
	Sheath dehiscence		1 (1.9)
	ITU/HDU admission		1 (1.9)
	VAC dressing		2 (3.7)
	Return to theatre		4 (7.4)

For peer review only

For peer review only - http://bmjopen.bmj.com/site/about/guidelines.xhtml

The incidence of infection per cancer centre was variable ranging from 6.3% - 41.2%. A funnel plot was constructed for each centre using the SSI incidence per centre, Figure 1.

Further review of the centre that appears to be an outlier (Figure 1, red triangle) demonstrates no evidence that the threshold for diagnosis of SSI was any lower at this centre. Wound swabs yielded growth of micro-organisms in 13/14 cases. However, 4/14 wound infections in this group were not prescribed antibiotics. There was no excess of pre-operative comorbidities in this subgroup of patients. However, there was a statistically significant difference in the radicality score of those with infection from this centre in comparison to the other 11 centres, with a mean score of 4.29 vs 2.38, p=0.0194.

Predictors of SSI

Univariate analysis suggested that higher BMI, ASA, diabetes requiring insulin, use of a drain, choice of antiseptic for skin preparation and skin closure were associated with significant increase in SSI risk (p<0.05), Table 4. After accounting for other variables, higher BMI was associated with having a greater risk of infection, with the odds of infection increasing by on average 8% (95% CI 3% to 14%) for each extra unit of BMI. Insertion of a wound drain and wound closure with staples rather than suture were all associated with having an increased risk of infection, Table 4. There was no evidence to suggest that ASA or using betadine rather than chlorhexidine skin preparation were associated with an increased infection rate, after taking into account the other variables.

For peer review only - http://bmjopen.bmj.com/site/about/guidelines.xhtml

Table 4: Predictors of SSI

Univariate logistic regression			
**Type of risk factor**	**Variable**	**OR (95% CI)**	**p-value**
Non-modifiable prior to surgery	BMI (per kg/m^2)	1.09(1.04 to 1.13)	<0.001
	Age (per year)	1.01(0.99 to 1.04)	0.236
	ASA 3 or more(v less than 3 or missing)	1.73(0.93 to 3.22)	0.083
	Albumin (per g/l)	0.98(0.93 to 1.02)	0.309
	Figo Stage (per unit)	1.05(0.76 to 1.45)	0.773
	Diabetic requiring insulin (vs not requiring insulin for glycaemic control)	5.00(1.61 to 15.5)	0.005
	Neoadjuvant chemotherapy (vs none)	0.50(0.22 to 1.17)	0.110
	Cancer type<br>Endometrium (vs ovarian)<br>Benign (vs ovarian)<br>Cervical (vs ovarian)<br>Other (vs ovarian)	 1.48(0.72 to 3.04)<br>0.68(0.08 to 5.64)<br>0.87(0.36 to 2.10)<br>0.73(0.16 to 3.34)	 0.291<br>0.722<br>0.752<br>0.682
Non-modifiable surgical risk factor	Surgical radicality score (per unit)	1.02(0.89 to 1.19)	0.718
	Clean-contaminated (vs clean)	2.26(0.67 to 7.66)	0.189
	Incision site<br>Above umbilicus (vs transverse)<br>Below umbilicus (vs transverse)	 1.64(0.36 to 7.39)<br>2.15(0.46 to 10.19)	 0.519<br>0.333
Modifiable at time of surgery	Chlorhexadine skin preparation (vs betadine)	0.54(0.30 to 0.97)	0.039
	Wound drain (vs none)	3.23(1.68 to 6.19)	<0.001
	Subcutaneous fat stitch (vs none)	1.50(0.76 to 2.95)	0.245
	Staples (vs subcutaneous suture)	3.58(1.84 to 6.97)	<0.001
	Prophylactic post-op antibiotics (vs none/missing)	1.69(0.86 to 3.31)	0.126
**Multivariate logistic regression**			
**Type of risk factor**	**Variable**	**OR (95% CI)**	**p-value**
Non-modifiable prior to surgery	BMI (per kg/m^2)	1.08(1.03 to 1.14)	0.001
	ASA (per unit)	1.01(0.48 to 2.11)	0.989
	Diabetic requiring insulin (vs not requiring insulin for glycaemic control)	3.34(0.90 to 12.39)	0.071
Modifiable at time of surgery	Chlorhexadine (vs betadine)	0.58(0.30 to 1.134)	0.109
	Drain (vs none)	2.92(1.41 to 6.04)	0.004
	Staples (vs SC suture)	3.13(1.50 to 6.56)	0.002

Sequelae

Median length of post-operative hospital stay was 5 days (Range 1–71, IQR 4-8). In those that developed infection, there was clinical delay in discharge in 18/54 (33.3%). In the 37 patients with SSI who required adjuvant chemotherapy or radiotherapy, 11 (29.7%) experienced delay in commencing therapy directly or indirectly as a result of infection.

For peer review only

For peer review only - http://bmjopen.bmj.com/site/about/guidelines.xhtml

Approximately half of all infections were diagnosed following discharge and 5 (1.5%) patients necessitated readmission to hospital following diagnosis of infection. 30 (55.6%) patients had evidence of skin dehiscence, 14 of which required wound packing. As a result of wound complications; 4 patients returned to theatre, 1 patient was admitted to level 2/3 care and 2 patients required negative pressure wound therapy.

Discussion

Modelled on the highly successful trainee-led research collaboratives in other surgical specialties, the surgical gynaecological research network (SGRN) was formed in 2015 to address the paucity of clinical research in gynaecological cancer surgery. Our study provides a snapshot of current practice of the pre-, intra and post-operative prevention and management of surgical site infection in women undergoing open gynaecological oncology surgery across the UK.

Our study demonstrates that SSI is highly prevalent within the gynaecological oncology population who undergo laparotomy with an incidence of 15.9%; ten times higher than the incidence reported in the NHS Surgical Site Infection Surveillance Programme for women undergoing abdominal hysterectomy in 2015/2016 [10]. The high burden of complications is likely to reflect a combination of patient, surgical and disease-specific factors with 90.3% of patients undergoing clean-contaminated or contaminated procedures. Unsurprisingly, infection is prevalent in patients with a high BMI, which perhaps explains the greater incidence observed in those within the endometrial cancer subgroup and in those with diabetes, given shared aetiological factors. Cancer and its treatments can result in immune deficiency making cancer patients in general undergoing surgery a high risk group, but in particular gynaecology oncology patients, with the contaminated nature and complexity of the procedures required [11].

In a small proportion of patients, SSI can be severe and life threatening, necessitating return to theatre or advanced wound care and prolonged hospital stays. Although no qualitative data was collected from patients during this observational study, it may be inferred that SSI has the capability of contributing towards significant impact on a patient's physical and perhaps psychological recovery.

Our study provides evidence that, not only does SSI result in prolonged hospital admission, but also delays time to delivery of adjuvant cancer treatments. This has the potential to impact upon response to treatment and potentially overall cancer specific survival [11–13]. However, additional data is needed to evaluate this further.

Health care associated infections can cause substantial morbidity as well as healthcare costs associated with management of complications and prolonged hospital admissions [14,15]. It is known that the additional cost of treating a surgical site complication ranges from £2500-£14000 [16]. It is estimated that the treatment of SSI and their sequelae costs the NHS over £700m annually[17]. The ancillary costs from time off work, reduced health related quality of life, dissatisfaction in patients, and litigation costs for the healthcare provider have been explored less extensively, but available evidence confirms a detrimental effect[18].

For peer review only - http://bmjopen.bmj.com/site/about/guidelines.xhtml

In this study, infection occurred in patients even where compliance with local infection prophylaxis standards were high. At a time when many NHS trusts are keen to invest in enhanced recovery programmes to encourage a speedy and proactive approach to recovery for patients, this audit demonstrates a need for effective management of wounds to minimise the incidence of wound infection and provide effective management rapidly when it does occur. Our group intends to develop and trial a stratified care bundle to reduce delay to adjuvant treatment associated with SSI.

Strengths and Limitations

To the best of our knowledge, this study is the first to comprehensively review current practice surrounding the prevention and management of SSI in open gynaecological cancer surgery. The UKGOSOC study found a slightly lower wound complication rate of 9.6% across all types of gynaecological cancer surgery in ten UK gynaecological cancer centres [1]. However, this study included patients undergoing both laparoscopic and open surgery. Laparoscopic surgery has previously been shown to have lower incidence of post-operative complications such as wound infection [19–21]. We have therefore chosen to examine care practices in women undergoing open surgery specifically.

The strength of our review of practice is that the data were prospectively collected using standardised data collection forms at 12 participating centres from around the UK. As this was a review of practice, data from all consecutive surgeries were included as patient recruitment was not required. Despite our use of a standardised proforma and the use of a pre-agreed definition of SSI, there remained a potential for variation in its interpretation from site to site. This may have contributed to the outlier unit with the disproportionately high rate of SSI. However, this type of variation is likely to also reflect that seen in day-to-day practice. Twelve of the 44 units which offer tertiary level gynaecological cancer surgery participated in this review. No units from the South West or Wales participated. The inclusion of these units together with a longer data collection window may have led to a larger sample, allowing the identification of further potential risk factors for SSI and highlighting additional practices that may reduce wound infection rates. The identification of risk factors and good practice points will enable us to develop a tool to personalise patient care plans with the aim of reducing surgical site infection.

Conclusions

This trainee-led collaborative initiative demonstrates the need to determine whether addressing modifiable risk factors can prevent SSI in gynaecological cancer surgery. Ultimately, a reduction in delayed discharge and subsequent delays in the commencement of adjuvant treatment could result in improved patient outcomes. With the support of various stakeholders including the British Gynaecological Cancer Society, National Cancer Research Institute, the Royal College of Surgeons and the Cochrane Wounds Group, we have laid the groundwork for future clinical trials in SSI prevention in gynaecological oncology.

For peer review only - http://bmjopen.bmj.com/site/about/guidelines.xhtml

References

1 Iyer R, Gentry-Maharaj A, Nordin A, *et al.* Predictors of complications in gynaecological oncological surgery: a prospective multicentre study (UKGOSOC—UK gynaecological oncology surgical outcomes and complications). *Br J Cancer* 2015;**112**:475–84. doi:10.1038/bjc.2014.630

2 NICE Clinical Guidelines. Surgical site infections: prevention and treatment. 2008. https://www.nice.org.uk/guidance/cg74/resources/surgical-site-infections-prevention-and-treatment-pdf-975628422853

3 Chief Medical Officer and Chief Nursing Officer. Surveillance of Healthcare Associated Infections. 2003. http://webarchive.nationalarchives.gov.uk/20130107105354/http://www.dh.gov.uk/prod_consum_dh/groups/dh_digitalassets/@dh/@en/documents/digitalasset/dh_4013410.pdf

4 Public Health England. Surveillance of Surgical Site Infections in NHS hospitals in England: 2016-2017. 2017. https://www.gov.uk/government/uploads/system/uploads/attachment_data/file/666465/SSI_annual_report_NHS_hospitals_2016-17.pdf

5 Dripps RD. New classification of physical status. *Anesthesiology* 1963;**24**:111.

6 The new CDC Guidelines on Infection Control. *Infect Control*;**3**:12–5.http://www.ncbi.nlm.nih.gov/pubmed/6276317 (accessed 16 Jan 2018).

7 Aletti GD, Dowdy SC, Podratz KC, *et al.* Relationship among surgical complexity , short-term morbidity , and overall survival in primary surgery for advanced ovarian cancer. *Obstet Gynecol* 2007;:1–7. doi:10.1016/j.ajog.2007.10.495

8 Horan TC, Andrus M, Dudeck MA. CDC/NHSN surveillance definition of health care-associated infection and criteria for specific types of infections in the acute care setting. *Am J Infect Control* 2008;**36**:309–32. doi:10.1016/j.ajic.2008.03.002

9 Public Health England. Protocol for the Surveillance of Surgical Site Infection. 2013. http://www.hpa.org.uk/webc/HPAwebFile/HPAweb_C/1194947388966

10 Public Health England. Surveillance of Surgical Site Infections in NHS hospitals in England. 2016. https://www.gov.uk/government/uploads/system/uploads/attachment_data/file/577418/Surgical_site_infections_NHS_hospitals_2015_to_2016.pdf

11 Tran CW, McGree ME, Weaver AL, *et al.* Surgical site infection after primary surgery for epithelial ovarian cancer: Predictors and impact on survival. *Gynecol Oncol* 2015;**136**:278–84. doi:10.1016/j.ygyno.2014.12.007

12 Haruki K, Shiba H, Fujiwara Y, *et al.* Negative impact of surgical site infection on long-term outcomes after hepatic resection for colorectal liver metastases. *Anticancer Res* 2013;**33**:1697–

For peer review only - http://bmjopen.bmj.com/site/about/guidelines.xhtml

703.http://www.ncbi.nlm.nih.gov/pubmed/23564818 (accessed 16 Jan 2018).

13 Buettner S, Ethun CG, Poultsides G, *et al.* Surgical Site Infection Is Associated with Tumor Recurrence in Patients with Extrahepatic Biliary Malignancies. *J Gastrointest Surg* 2017;**21**:1813–20. doi:10.1007/s11605-017-3571-2

14 Wilson APR. Surgical wound infection as a performance indicator: agreement of common definitions of wound infection in 4773 patients. *BMJ* 2004;**329**:720–0. doi:10.1136/bmj.38232.646227.DE

15 Mangram AJ, Horan TC, Pearson ML, *et al.* Guideline for Prevention of Surgical Site Infection, 1999. Centers for Disease Control and Prevention (CDC) Hospital Infection Control Practices Advisory Committee. *Am J Infect Control* 1999;**27**:97–132.http://www.ncbi.nlm.nih.gov/pubmed/10196487 (accessed 16 Jan 2018).

16 NHS Clinical Comissioning Groups. NICE support for commissioning for surgical site infection. 2013;:1–16.

17 Adams-Howell P, Bhabra M, Enright M, *et al.* Under the Knife: Taking a zero tolerance approach to preventable surgical site infections in UK hospitals. 2011;:1–12.

18 Perencevich E Cosgrove S, Guadagnoli E, Meara E, Platt R SK, Perencevich EN Cosgrove SE, Guadagnoli E, Meara E, Platt R SKE. Health and economic impact of surgical site infections diagnosed after hospital discharge. *Emerg Infect Dis* 2003;**9**:196–203.

19 Mahdi H, Gojayev A, Buechel M, *et al.* Surgical Site Infection in Women Undergoing Surgery for Gynecologic Cancer. *Int J Gynecol Cancer* 2014;**24**:779–86. doi:10.1097/IGC.0000000000000126

20 Walker JL, Piedmonte MR, Spirtos NM, *et al.* Laparoscopy compared with laparotomy for comprehensive surgical staging of uterine cancer: Gynecologic Oncology Group Study LAP2. *J Clin Oncol* 2009;**27**:5331–6. doi:10.1200/JCO.2009.22.3248

21 Park J-Y, Kim D-Y, Kim J-H, *et al.* Laparoscopic versus open radical hysterectomy in patients with stage IB2 and IIA2 cervical cancer. *J Surg Oncol* 2013;**108**:63–9. doi:10.1002/jso.23347

For peer review only

For peer review only - http://bmjopen.bmj.com/site/about/guidelines.xhtml

Figure Legends

Figure 1: Funnel plot of SSI by UK cancer centre: Funnel plot of the number of reported surgical site infections for each gynaecological cancer unit versus effective sample size for each unit. The funnels correspond to 95% confidence limits (dashed lines). The outlier unit is denoted by a triangle, whilst units with SSI incidence within the 95% confidence limits are marked with a circle.

For peer review only

For peer review only - http://bmjopen.bmj.com/site/about/guidelines.xhtml

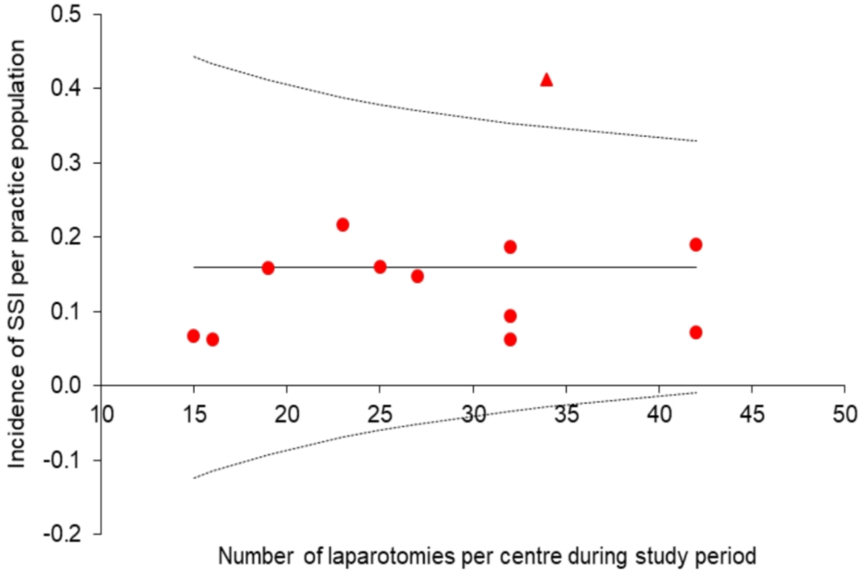

Figure 1: Funnel plot of SSI by UK cancer centre: Funnel plot of the number of reported surgical site infections for each gynaecological cancer unit versus effective sample size for each unit. The funnels correspond to 95% confidence limits (dashed lines). The outlier unit is denoted by a triangle, whilst units with SSI incidence within the 95% confidence limits are marked with a circle.

Supplementary Table A:

Term	Definition	Criteria (Any 1 of criteria below)
Superficial incisional infection	SSI within 30 days of surgery involving the skin or subcutaneous tissue of the incision	1. Purulent drainage from incision<br>2. Positive microbiological culture/pus cells present on swab from incision<br>3. Two of:<br>a. pain or tenderness<br>b. swelling<br>c. redness<br>4. the superficial incision is deliberately opened by a surgeon to manage the infection<br>5. clinician diagnoses a superficial incisional infection.
Deep incisional infection:	SSI involving the deep tissues (i.e. fascial and muscle layers) that occurs within 30 days of surgery<br>.	1. Purulent drainage from the deep incision<br>2. Positive microbiological culture/pus cells present on swab from fluid from deep incision<br>3. Spontaneous dehiscence/opening of wound by surgeon AND patient has at least 1 of:<br>a. fever (>38°C)<br>b. localized pain or tenderness<br>4. Radiologically, histologically or surgically diagnosed abscess involving the deep incision.<br>5. Clinician diagnoses a deep incisional SSI
Patient reported SSI	SSI reported by patient to have occurred within 30 days of surgery	1. Discharge pus AND antibiotics prescribed<br>2. Clinical signs* AND dehiscence<br>3. Clinical signs* AND antibiotics prescribed<br><br>*clinical signs – at least 2 of the following must be present: pain, heat, redness or swelling

For peer review only

Supplementary Table B: Modified surgical complexity scoring system based upon complexity and number of surgical procedures performed

Adapted from [10]

**Procedure**	**Points**
Total Hysterectomy- Bilateral Salpingo-Oophorectomy (TH-BSO)	1
Omentectomy	1
Pelvic lymphadenectomy	1
Paraaortic lymphadenectomy	1
Pelvic peritoneum stripping	1
Abdominal peritoneum stripping	1
Rectosigmoidectomy EEA	3
Large bowel resection	2
Diaphragm stripping/resection	2
Splenectomy	2
Liver resection	2
Small bowel resection	1
Resection of tail of pancreas	5
Cholecystectomy	5
Partial gastrectomy	5

Complexity score groups

1. Standard/ (low) ≤3

2. Intermediate 4-8

3. Ultra-radical (high) ≥8

For peer review only

Revised Standards for Quality Improvement Reporting Excellence (SQUIRE 2.0) September 15, 2015

Text Section and Item Name	Section or Item Description	
Notes to authors	The SQUIRE guidelines provide a framework for reporting new knowledge about how to improve healthcare<br>The SQUIRE guidelines are intended for reports that describe system level work to improve the quality, safety, and value of healthcare, and used methods to establish that observed outcomes were due to the intervention(s).<br>A range of approaches exists for improving healthcare. SQUIRE may be adapted for reporting any of these.<br>Authors should consider every SQUIRE item, but it may be inappropriate or unnecessary to include every SQUIRE element in a particular manuscript.<br>The SQUIRE Glossary contains definitions of many of the key words in SQUIRE.<br>The Explanation and Elaboration document provides specific examples of well-written SQUIRE items, and an in-depth explanation of each item.<br>Please cite SQUIRE when it is used to write a manuscript.	As you review the manuscript, place a checkmark in this column for each SQUIRE item that is appropriately addressed in the manuscript. Remember that not every item is necessary in every manuscript.
Title and Abstract		
1. Title	Indicate that the manuscript concerns an initiative to improve healthcare (broadly defined to include the quality, safety, effectiveness, patient-centeredness, timeliness, cost, efficiency, and equity of healthcare)	***Yes, inclusion in the title of audit and service evaluation i.e. methods through which healthcare is improved.***

For peer review only

2. Abstract	a. Provide adequate information to aid in searching and indexing<br>b. Summarize all key information from various sections of the text using the abstract format of the intended publication or a structured summary such as: background, local problem, methods, interventions, results, conclusions	***a. Keywords for indexing included: gynaecological oncology, wound management, surgical site infection, medical education,***<br>***b. Structured abstract as per BMJ Open guidance***
Introduction	*Why did you start?*	
3. Problem Description	Nature and significance of the local problem	***Pg 4, Lines 85-111***
4. Available knowledge	Summary of what is currently known about the problem, including relevant previous studies	***Pg 4, Lines 90-101***
5. Rationale	Informal or formal frameworks, models, concepts, and/or theories used to explain the problem, any reasons or assumptions that were used to develop the intervention(s), and reasons why the intervention(s) was expected to work	***Pg 4, Lines 102-106***
6. Specific aims	Purpose of the project and of this report	***Pg 4, Lines 107-111***
Methods	*What did you do?*	
7. Context	Contextual elements considered important at the outset of introducing the intervention(s)	***Pg 4, Lines 113-115***
8. Intervention(s)	Description of the intervention(s) in sufficient detail that others could reproduce it<br>Specifics of the team involved in the work	***Pg 5, Lines 120-122, Contributions, Pg 3 Lines 79-81***
9. Study of the Intervention(s)	Approach chosen for assessing the impact of the intervention(s)<br>Approach used to establish whether the observed outcomes were due to the intervention(s)	***Pg 5, Lines 123-136***<br>***n/a***
10. Measures	Measures chosen for studying processes and outcomes of the intervention(s), including rationale for choosing them, their operational definitions, and their validity and reliability<br>Description of the approach to the ongoing assessment of contextual elements that contributed to the success, failure, efficiency, and cost	***Pg 5, Lines 123-136***<br>***n/a***

For peer review only

	Methods employed for assessing completeness and accuracy of data	***Pg 5, Lines 137 – Lines 140***
11. Analysis	Qualitative and quantitative methods used to draw inferences from the data<br>Methods for understanding variation within the data, including the effects of time as a variable	***Pg 5, Lines 142-148***<br><br>***Pg 5, Lines 145-148***
12. Ethical Considerations	Ethical aspects of implementing and studying the intervention(s) and how they were addressed, including, but not limited to, formal ethics review and potential conflict(s) of interest	***Pg 4, Lines 116-119***
Results	*What did you find?*	
13. Results	Initial steps of the intervention(s) and their evolution over time (*e.g.*, time-line diagram, flow chart, or table), including modifications made to the intervention during the project<br>Details of the process measures and outcome<br>Contextual elements that interacted with the intervention(s)<br>Observed associations between outcomes, interventions, and relevant contextual elements<br>Unintended consequences such as unexpected benefits, problems, failures, or costs associated with the intervention(s).<br>Details about missing data	***Pg 6, Lines 155-159***<br><br>***Pg 6, 8, 12, Table 3***<br>***Pg 8, 10, 11, Table 1 & 2***<br>***Pg 10, Table 4***<br><br>***n/a***<br><br>***Pg 6, Line 156-157***
Discussion	*What does it mean?*	
14. Summary	Key findings, including relevance to the rationale and specific aims<br>Particular strengths of the project	***Lines 61-68, Pg 12***<br>***Pg 13, Lines 277-286***
15. Interpretation	Nature of the association between the intervention(s) and the outcomes<br>Comparison of results with findings from other publications<br>Impact of the project on people and systems<br>Reasons for any differences between observed and anticipated outcomes, including the influence of context<br>Costs and strategic trade-offs, including opportunity costs	***Pg 12-13, Lines 241-275***<br>***Pg 13. Lines 278-282***<br><br>***Pg 12, Lines 256-275***<br>***Pg 12, Lines 249-255***<br>***n/a***
16. Limitations	Limits to the generalizability of the work<br>Factors that might have limited internal validity such as confounding, bias, or imprecision in the design, methods, measurement, or analysis<br>Efforts made to minimize and adjust for limitations	***Pg 13, Lines 289-293, Lines 66-68***<br>***Pg 13, Lines 291-295***<br><br>***Pg 13, Lines 286-291***

For peer review only

17. Conclusions	Usefulness of the work<br>Sustainability<br>Potential for spread to other contexts<br>Implications for practice and for further study in the field<br>Suggested next steps	***Pg 13, Lines 297-302***<br>***Not assessable at this stage in the project***<br>***Pg 13, Lines 297-302***<br>***Pg 13, Lines 297-302***
Other information		
18. Funding	Sources of funding that supported this work. Role, if any, of the funding organization in the design, implementation, interpretation, and reporting	***Pg 3, Lines 70-73***

For peer review only

BMJ Open

IMPACT OF SURGICAL SITE INFECTION (SSI) FOLLOWING GYNAECOLOGICAL CANCER SURGERY IN THE UNITED KINGDOM: A TRAINEE-LED MULTICENTRE AUDIT AND SERVICE EVALUATION

Journal:	*BMJ Open*
Manuscript ID	bmjopen-2018-024853.R1
Article Type:	Research
Date Submitted by the Author:	20-Oct-2018

Complete List of Authors:	O'Donnell, Rachel; Northern Gynaecological Oncology Centre; Surgical Gynaecological Oncology Research Network<br>Angelopoulos, Georgios; Surgical Gynaecological Oncology Research Network; James Cook University Hospital<br>Beirne, James; Surgical Gynaecological Oncology Research Network; Northern Ireland Centre for Gynaecological Oncology, Belfast City Hospital<br>Biliatis, Ioannis; Surgical Gynaecological Oncology Research Network; Royal Marsden NHS Foundation Trust,<br>Bolton, Helen; Surgical Gynaecological Oncology Research Network; Addenbrooke's Hospital<br>Bradbury, Melissa; Surgical Gynaecological Oncology Research Network; Northern Gynaecological Oncology Centre<br>Craig, Elaine; Surgical Gynaecological Oncology Research Network; Northern Ireland Centre for Gynaecological Oncology, Belfast City Hospital<br>Gajjar, Ketan; Surgical Gynaecological Oncology Research Network; Addenbrooke's Hospital<br>Mackintosh, Michelle; Surgical Gynaecological Oncology Research Network, ; St Mary's Hospital, Gynaecological Oncology<br>MacNab, Wendy; Surgical Gynaecological Oncology Research Network; Glasgow Royal Infirmary<br>Madhuri, Thumuluru Kavitha; Surgical Gynaecological Research Network; Royal Surrey County Hospital NHS Foundation Trust, Department of Gynaecological Oncology<br>McComiskey, Mark; Surgical Gynaecological Oncology Research Network; St Mary's Hospital, Gynaecological Oncology<br>Myriokefalitaki, Eva; Surgical Gynaecological Oncology Research Network; University Hospitals of Leicester NHS Trust, Gynaecological Oncology<br>Newton, Claire; Surgical Gynaecological Oncology Research Network; Saint Bartholomew's Hospital, Gynaecological Oncology<br>Ratnavelu, Nithya; Surgical Gynaecological Oncology Research Network; Northern Gynaecological Oncology Centre<br>Taylor, Sian; Surgical Gynaecological Oncology Research Network; Liverpool Womens NHS Foundation Trust, Gynaecological Oncology<br>Thangavelu, Amudha; Surgical Gynaecological Oncology Research Network; Nottingham University Hospitals NHS Trust, Gynaecological

	Oncology<br>Rhodes, Sarah; Centre for Biostatistics, Faculty of Biology, Medicine and Health<br>Crosbie, Emma; Surgical Gynaecological Oncology Research Collaboration; University of Manchester Faculty of Biology, Medicine and Health, Gynaecological Oncology<br>Edmondson, Richard; Surgical Gynaecological Oncology Research Network; University of Manchester Faculty of Biology, Medicine and Health, Gynaecological Oncology<br>Wan, Yee-Loi; University of Manchester Faculty of Biology, Medicine and Health, Gynaecological Oncology
**Primary Subject Heading**:	Obstetrics and gynaecology
Secondary Subject Heading:	Surgery
Keywords:	WOUND MANAGEMENT, SURGERY, Gynaecological oncology < GYNAECOLOGY, surgical site infection, noscomial infection, hospital acquired infection

SCHOLARONE™ Manuscripts

IMPACT OF SURGICAL SITE INFECTION (SSI) FOLLOWING GYNAECOLOGICAL CANCER SURGERY IN THE UNITED KINGDOM: A TRAINEE-LED MULTICENTRE AUDIT AND SERVICE EVALUATION

Authors

Rachel L O'Donnell 1,2, Georgios Angelopoulos 1,3, James P. Beirne 1,4, Ioannis Biliatis 1,5, Helen Bolton 1,6, Melissa Bradbury 1,2, Elaine Craig 1,4, Ketankumar Gajjar 1,6, Michelle L MacKintosh 1,7, Wendy MacNab 1,8, Thumuluru K. Madhuri Madhuri 1,9, Mark McComiskey 1,7, Eva Myriokefalitaki 1,10, Claire L Newton 1,11, Nithya Ratnavelu 1,2, Siân E. Taylor 1,12, Amudha Thangavelu 1,13, Sarah A. Rhodes 14, Emma J Crosbie 1,15, Richard J Edmondson 1,15, Yee-Loi Wan 1,15

1. Surgical Gynaecological Oncology Research Network
2. Northern Gynaecological Oncology Centre, Gateshead Foundation NHS Trust, Gateshead NE9 6SX
3. James Cook Hospital, Marton Road, Middlesbrough, TS4 3BW
4. Northern Ireland Centre for Gynaecological Cancer, Belfast City Hospital, Lisburn Road, Belfast, BT9 7AB
5. Royal Marsden Hospital, Fulham Road, London, SW3 6JJ
6. Addenbrooke's Hospital, Hills Road, Cambridge, CB2 0QQ
7. St Mary's Hospital, Manchester University Hospitals NHS Foundation Trust, Manchester Academic Health Science Centre; Oxford Road, Manchester, M13 9WL
8. Glasgow Royal Infirmary, Castle Street, Glasgow, G4 0SF
9. Royal Surrey County Hospital, Egerton Rd, Guildford GU2 7XX
10. University Hospital Leicester, Infirmary Square, Leicester LE1 5WW
11. St Bartholomew's Hospital, West Smithfield, London EC1A 7BE
12. Liverpool Women's Hospital, Crown St, Liverpool L8 7SS
13. Nottingham University Hospital, Hucknall Rd, Nottingham NG5 1PB
14. Centre for Biostatistics, Division of Population Health, Health Services Research and Primary Care, University of Manchester, Oxford Road, Manchester M13 9PL
15. Division of Cancer Sciences, Faculty of Biology, Medicine and Health, University of Manchester, M13 9WL

Corresponding author: Y Louise Wan

Corresponding author email address: louise.wan@manchester.ac.uk

For peer review only - http://bmjopen.bmj.com/site/about/guidelines.xhtml

Abstract

Objectives

Surgical site infection (SSI) complicates 5% of all surgical procedures in the United Kingdom (UK), and is not only a major cause of post-operative morbidity, but also a substantial drain on healthcare resources. Little is known about the incidence of SSI and its consequences in women undergoing surgery for gynaecological cancer. Our aim was to perform the first national audit of SSI following gynaecological cancer surgery through the establishment of a UK-wide trainee-led research network.

Design and setting

In a prospective audit, we collected data from all women undergoing laparotomy for suspected gynaecological cancer at 12 specialist oncology centres in the UK during an eight-week period in 2015. Clinicopathological data were collected and wound complications and their sequelae were recorded during the 30 days following surgery.

Results

In total, 339 women underwent laparotomy for suspected gynaecological cancer during the study period. A clinical diagnosis of SSI was made in 54 (16%) women. 92.6% (50/54) of women with SSI had prolonged hospital stays and 11/37 (29%) had their adjuvant treatment delayed or cancelled. Multivariate analysis found BMI was the strongest risk factor for SSI [OR 1.08 (95% CI 1.03 to 1.14) per 1kg/m^2 increase in BMI, (p=0.001)]. Wound drains [OR 2.92(95% CI 1.41 to 6.04), p=0.004] and staple closure [OR 3.13 (95% CI 1.50 to 6.56), p=0.002] were also associated with increased risk of SSI.

Conclusions

SSI is common in women undergoing surgery for gynaecological cancer leading to delays in discharge and adjuvant treatment. Resultant delays in adjuvant treatment may impact cancer-specific survival rates. Modifiable factors, such as choice of wound closure material offer opportunities for reducing SSI and reducing morbidity in these women. There is a clear need for new trials in SSI prevention in this patient group; our trainee-led initiative provides a platform for their successful completion.

Abstract word count: 299

Strengths & Limitations of this study

- This study documents real-world clinical practice in the prevention and management of surgical site infection (SSI) in women undergoing open gynaecological cancer surgery in the UK.

For peer review only - http://bmjopen.bmj.com/site/about/guidelines.xhtml

- Data were collected prospectively and consecutively without exclusions according to comorbidities or treatment from all women undergoing gynaecological cancer surgery at 12 UK centres, encompassing the various national care settings (except Wales).
- Data relating to direct and indirect health care costs of SSIs were not available.
- Further longitudinal data examining the cancer specific outcomes of this cohort are not currently available.

Funding Statement

YLW was funded by the Wellcome Trust as part of a Wellcome Trust Clinical Research Fellowship (101714/Z/13/Z).

EJC is a National Institute for Health Research (NIHR) Clinician Scientist (NIHR-CS-012-009) and is supported by the NIHR Manchester Biomedical Research Centre

Patient involvement was funded by a NIHR North West Research Design Service Patient and Public Involvement Bursary. Neither funder was involved in the planning or execution of the study or the drafting of the manuscript.

Competing interests

No competing interests.

Contributions

RLOD, YLW, MB, TKM, AT, MLM, MM, EJC and RJE conceived and designed the study. Data was collected by RLOD, NR, GA, MB, IB, HB, JPB, EC, KG, WM, TKM, MLM, MM, EM, CLN, NR, SET and AT and analysed by RLOD, MM, TKM, SAR and YLW. The article was drafted by RLOD and YLW and revised and approved by all authors. All authors agree to be accountable for all aspects relating to the accuracy or integrity of the work.

Data sharing

Original data are available from the corresponding author on request.

Article word count

2891

For peer review only

Introduction

Gynaecological cancers collectively account for 11% of all cancers affecting women in the UK. Surgery remains the mainstay of treatment and despite an increase in the use of minimally invasive techniques, a large proportion of patients still require laparotomy. For many women, their treatment will involve a hysterectomy with an associated high risk of surgical site infection (SSI)due to the inherently contaminated nature of procedures involving entry into the genital tract.

Although most women undergoing laparotomy can expect their wound to heal without any problem, there is a lack of high-quality data in gynaecological oncology to indicate the incidence and consequences of SSI. A study by Iyer et al, which aimed to identify predictors of complications in women undergoing surgery for gynaecological malignancy, describes Grade II-IV infections in 27.8% of all gynaecological surgery cases accounting for 31% of all complications [1]. Although this study identified predictors of complications, the consequences of such complications were not assessed. The need to identify preventable complications coupled with effective interventions remains. The importance of SSI prevention, across all surgical specialities, has also been acknowledged by the UK government with the publication of various national guidelines outlining care bundles with recommended interventions during the pre-operative, intraoperative and postoperative phases of the patient journey[2,3]. This has led to a downward trend in the incidence of SSIs in most types of surgery [4]. However, worryingly, there has been a year on year rise in SSI related to abdominal hysterectomies since 2014 [4].

Further research into understanding the direct and indirect effects of SSI upon the gynaecological oncology population and the additional interventions that could reduce surgical site infections is, therefore, urgently needed. An innovative UK-wide multi-centre collaborative of trainees and consultants with an interest in clinical research in gynaecological oncology was established in 2015 to develop and implement evidence-based interventions to improve surgical care for women undergoing gynaecological cancer surgery.

The primary objective of this study was to define the incidence of SSI in the UK gynaecological oncology population undergoing laparotomy and record standard practices. The secondary objectives were to identify risk factors associated with infection and assess the potential impact upon length of stay and delay to adjuvant cancer treatment. This study also aimed to provide baseline data to develop a modified enhanced recovery programme for women undergoing gynaecological cancer surgery.

Methods

This prospective multi-centre audit of all consecutive women undergoing laparotomy for suspected or confirmed gynaecological malignancy was undertaken across 12 of the 44 UK tertiary centres during an 8-week period September - November 2015. Daily review of surgical cases was undertaken in each institute and cross referenced with theatre IT systems to ensure all subjects were included. Advice was sought from local ethics committees, who confirmed that this study was exempt from ethical review as the research was limited to the

For peer review only - http://bmjopen.bmj.com/site/about/guidelines.xhtml

secondary use of fully anonymised information previously collected in the course of normal care without the intention to use it for research at the time of collection.

Local audit leads were recruited via the British Gynaecological Cancer Society Trainees group and the Tomorrows Leaders in Surgical Research workshops. These local leads co-ordinated the prospective collection and coding of anonymised data using a standardised data-collection tool developed in line with NICE guidance [2]. Additional information including patient demographics, comorbidities, surgicopathological and wound complication data were also contemporaneously collected. All patients were categorised using the derived patient status risk index described by the American Society of Anaesthesiologists (ASA) [5]. Surgical complexity was classified using a modification of the surgical scoring system (Supplementary Table A) developed by the Mayo clinic [6] for ovarian cancer. Each individual procedure was given a score and the total score for the surgery was the sum of the individual scores. Surgeries were categorised into three groups of increasing complexity based on the overall score (Low - score ≤3; Intermediate – score 4-8; Radical - ≥8). In addition to the procedure, the duration of surgery and estimated blood loss (EBL) were also recorded.

The Centres for Disease Control and Prevention (CDC) definitions for superficial incisional, deep incisional and organ/space SSI were used to standardise diagnosis of SSI across all centres (Supplementary Table B) [7]. A modified definition was applied for patient reported SSI, as per the Public Health England Surgical Site Infection Surveillance programme protocol [8]. The impact of SSI on length of stay, return to theatre, use of negative pressure wound therapy, admission to intensive care and time to adjuvant chemo or radiotherapy was studied.

To ensure completeness of data and to capture events following discharge, all recorded interactions (i.e. telephone consultations/clinic visits/hospital records) with patients in the 30 days post-operatively were analysed. Data from patient reported infection following discharge as well as observed infection during admission were included in the analysis.

Statistical analysis

Descriptive analysis of clinico-pathological data was undertaken to characterise the cohort and the predictive value of each risk factor for SSI was separately assessed using univariable analysis by logistical regression or Chi^2 as appropriate. The variables to include in the logistic regression were based on the univariate analysis and known risk factors for SSI. To take into account variations in the length of time to last recorded contact, the incidence density was calculated using the number of cases in the numerator and the total number of days of patient follow-up (from inpatient surveillance) in the denominator giving the number of SSIs per 10 patient days of follow-up.

Patient involvement

No patients were directly involved in this research. However, the views of focus groups were sought regarding the most appropriate sequelae of SSI to measure. Patients are actively involved in the design of potential interventions to prevent SSIs and any future trials from our trainee-led initiative.

For peer review only - http://bmjopen.bmj.com/site/about/guidelines.xhtml

Results

Data Collection

During the prospective study, undertaken between 1st September 2015 and 1st November 2015, with follow-up until 1st December 2015, 12 UK tertiary centres submitted data for a total of 339 patients. Data were complete in 98.0% of fields. Median time until last patient encounter was 22 days (Interquartile range (IQR) 14-41 days). The final patient contact in the post-operative follow up period for most patients was at their outpatient review 4-6 weeks after surgery.

Participants

Baseline patient characteristics are shown in Table 1. The median patient age was 62 years (IQR 53–71) with a median body mass index (BMI) of 28.0 kg/m^2 (IQR 24.0 – 32.8). Pre-operative performance status varied with 62 (18.3%) patients classified as ASA 1; 166 (49.0%) as ASA 2; and 87 (25.7%) as ASA 3. 13% of patients were hypoalbuminaemic (albumin <35g/dL).

The majority of surgeries were undertaken as primary treatments (244, 72%) with 69 (20.4%) patients undergoing interval surgery after neoadjuvant chemotherapy for ovarian cancer. 22 (6.5%) patients underwent surgery for recurrent disease with palliative intent (from all primary sites). Of the 339 study patients, 288 (85.0%) had a diagnosis of cancer confirmed, the majority of whom, 200 (59%), had a diagnosis of ovarian cancer, Table 1.

For peer review only - http://bmjopen.bmj.com/site/about/guidelines.xhtml

Table 1: Patient Demographics

Characteristic	Cases (%) – unless otherwise stated
**Age**	n=339
Age (years), median (interquartile range)	62 (51-71)
**BMI**	n=323
BMI (kg/m^2), median (interquartile range)	28.0 (15.0 – 60.6)
ASA	n=315
1	62 (18.3)
2	166 (49.0)
3	87 (25.7)
Co-morbidity	n=334
Diabetes	34 (10.0)
Oral hypoglycaemic / insulin use	21 (6.2) / 13 (3.8)
Thyroid dysfunction	43 (12.7)
Immune defect	2 (0.6)
Pre-operative anaemia	34 (10.0)
Coagulopathy	9 (2.7)
Chronic kidney disease	15 (4.4)
Chronic lung disease	37 (10.9)
Current smoker	36 (10.6)
Other non-active malignancy	16 (4.7)
Diagnosis	n=337
Ovarian /Primary peritoneal carcinoma	200 (59.3)
Endometrial cancer	61 (18.1)
Cervical	9 (2.7)
Benign	51 (15.1)
Other non-gynaecological cancer	18 (5.3)
FIGO Stage	n=325
1	83 (25.5)
2	23 (7.1)
3	129 (39.7)
4	24 (7.4)
Not applicable	66 (20.3)
Surgical radicality	n=338
Standard (≤3)	265 (78.4)
Intermediate (4 - 8)	61 (18.0)
Ultra-radical (≥8)	13 (3.8)
**Procedure**	n=338
Hysterectomy	272 (80.2)
Oophorectomy/salpingectomy	305 (90.0)
Omentectomy	250 (73.7)
Pelvic lymph node dissection	67 (19.8)
Para-aortic lymph node dissection	35 (10.3)
Small bowel resection	19 (5.6)
Large bowel resection	36 (10.6)
Peritoneal stripping	55 (16.2)
Panniculectomy	3 (0.9)
Colostomy	15 (4.4)
Urostomy	2 (0.6)

Abbreviations. ASA: American Society of Anaesthesiologists; BMI: body mass index.

For peer review only - http://bmjopen.bmj.com/site/about/guidelines.xhtml

Infection prophylaxis

All patients were screened for MRSA pre-operatively, and colonisation with MRSA was confirmed in 2 patients who subsequently underwent pre-operative eradication. Infection prophylaxis policy varied between centres with variations in surgical skin preparation and prophylactic antibiotics. . 318/339 (93.8%) patients received intravenous antibiotics at induction of anaesthesia, of which 292/318 (91.8%) was in line with local microbiology policy. Of those who received prophylactic intra operative antibiotics, 23 (7.2%) received triple therapy, 85 (26.7%) received combined double therapy and 210 (66.0%) received single agent prophylaxis. The most frequently used antibiotic was co-amoxiclav, used alone or in combination in 236 (69.6%) patients. 306 (90.3%) surgeries were classified as clean-contaminated or contaminated.

Surgery

The majority of laparotomy incisions were midline and extended above the level of the umbilicus to the pubis. Median operating time was 150 minutes (IQR 110-191 minutes). The median surgical complexity score for the entire cohort was 2 (Range 1–17, IQR 2-3), Table 1. 74 (21.8%) patients had a radicality score of ≥4 which included 50 (15.6%) patients who underwent bowel resection, Table 1.

Post-operative wound management

Use of wound drains and skin closure techniques was highly variable and the incidence of drain use, skin closure and dressing used are shown in Table 2. Dressing choice was largely dependent upon NHS site, with little variation within each institution.

Table 2: Post-operative wound management

**Technique**		**Usage n (%)**		**Missing data (n)**
**Intra-abdominal drain**		59	17.4	7
**Subcutaneous tissue closure**		68	20.1	7
**Local anaesthetic wound catheter**		16	4.7	7
**Skin closure**	*Staples*	174	51.3	0
	Subcuticular monocryl	164	48.4	
	Other	1	0.3	
**Wound dressing**	*Mepore*	145	42.8	2
	Tegaderm	68	20.1	
	Opsite	59	17.4	
	Other	63	18.6	

Surgical site infections

Of the 339 patients included in the study, 54 (15.9%) were diagnosed with a surgical site infection by CDC criteria and 30 (8.8%) had wound dehiscence. 20 (5.9%) patients had dehiscence in the presence of infection. To take into account the variation in the length of follow up, incidence density was calculated; 0.5 cases of infection per 10 days of patient follow-up was seen. Severity of infection was variable with the incidence of various markers of severity shown in Table 3. Infection was most prevalent in patients undergoing laparotomy

For peer review only - http://bmjopen.bmj.com/site/about/guidelines.xhtml

For peer review only

for endometrial cancer, with an incidence of 21.3% in this subgroup. However, there was no significant difference in incidence of SSI by site of tumour origin, p=0.7358.

Causative micro-organisms were isolated from wound culture in only 32/54 cases (59.3%). The most prevalent culture result showing mixed growth predominantly of Escherichia Coli and/or other bowel commensals. Staphylococcus aureus was a reported cause of SSI in 4 cases in this series.

Table 3: Markers of SSI severity

Infection type and Severity n=54			**Cases (%) -unless otherwise stated**
**Infection**	Cultured organisms	*Positive culture*	32 (59.3)
	Time of infection	*Post-op admission*	24 (44.4.)
		Community	25 (46.3)
		Local hospital readmission	4 (7.4)
		Central hospital readmission	1 (1.9)
	Antibiotic route	*IV*	24 (44.4)
		PO	25 (46.3)
		None	6 (11.1)
	Length of course	*Days, median (range)*	7 (5 – 14)
**Severity**	Pyrexia	*Temp >38°C*	11 (20.4)
	WBC	*x10/L, median (interquartile range)*	10 (7 – 14)
	Cellulitis	*Clinical diagnosis*	24 (44.4)
	Skin dehiscence	*Clinical diagnosis*	30 (55.6)
		Treated with packing	14 (25.9)
		Managed without packing	16 (29.6)
	Sheath dehiscence		1 (1.9)
	ITU/HDU admission		1 (1.9)
	VAC dressing		2 (3.7)
	Return to theatre		4 (7.4)

For peer review only

For peer review only - http://bmjopen.bmj.com/site/about/guidelines.xhtml

The incidence of infection per cancer centre was variable ranging from 6.3% - 41.2%. A funnel plot was constructed for each centre using the SSI incidence per centre, Figure 1.

Further review of the centre that appears to be an outlier (Figure 1, red triangle) demonstrates no evidence that the threshold for diagnosis of SSI was any lower at this centre. There was no excess of pre-operative comorbidities in this subgroup of patients. However, there was a statistically significant difference in the radicality score of those with infection from this centre in comparison to the other 11 centres, with a mean score of 4.29 vs 2.38, $p=0.0194$.

Predictors of SSI

Univariate analysis suggested that higher BMI, ASA, diabetes requiring insulin, use of a drain, choice of antiseptic for skin preparation and skin closure were associated with significant increase in SSI risk ($p<0.05$), Table 4. After accounting for other variables, higher BMI was associated with having a greater risk of infection, with the odds of infection increasing by on average 8% (95% CI 3% to 14%) for each extra unit of BMI. Insertion of a wound drain and wound closure with staples rather than suture were all associated with having an increased risk of infection, Table 4. There was no evidence to suggest that ASA or using betadine rather than chlorhexidine skin preparation were associated with an increased infection rate, after taking into account the other variables.

For peer review only

For peer review only - http://bmjopen.bmj.com/site/about/guidelines.xhtml

Table 4: Predictors of SSI

Univariate logistic regression			
**Type of risk factor**	**Variable**	**OR (95% CI)**	**p-value**
Non-modifiable prior to surgery	BMI (per kg/m²)	1.09(1.04 to 1.13)	<0.001
	Age (per year)	1.01(0.99 to 1.04)	0.236
	ASA 3 or more(v less than 3 or missing)	1.73(0.93 to 3.22)	0.083
	Albumin (per g/l)	0.98(0.93 to 1.02)	0.309
	Figo Stage (per unit)	1.05(0.76 to 1.45)	0.773
	Diabetic requiring insulin (vs not requiring insulin for glycaemic control)	5.00(1.61 to 15.5)	0.005
	Neoadjuvant chemotherapy (vs none)	0.50(0.22 to 1.17)	0.110
	Cancer type<br>Endometrium (vs ovarian)<br>Benign (vs ovarian)<br>Cervical (vs ovarian)<br>Other (vs ovarian)	 1.48(0.72 to 3.04)<br>0.68(0.08 to 5.64)<br>0.87(0.36 to 2.10)<br>0.73(0.16 to 3.34)	 0.291<br>0.722<br>0.752<br>0.682
Non-modifiable surgical risk factor	Surgical radicality score (per unit)	1.02(0.89 to 1.19)	0.718
	Clean-contaminated (vs clean)	2.26(0.67 to 7.66)	0.189
	Incision site<br>Above umbilicus (vs transverse)<br>Below umbilicus (vs transverse)	 1.64(0.36 to 7.39)<br>2.15(0.46 to 10.19)	 0.519<br>0.333
Modifiable at time of surgery	Chlorhexadine skin preparation (vs betadine)	0.54(0.30 to 0.97)	0.039
	Wound drain (vs none)	3.23(1.68 to 6.19)	<0.001
	Subcutaneous fat stitch (vs none)	1.50(0.76 to 2.95)	0.245
	Staples (vs subcutaneous suture)	3.58(1.84 to 6.97)	<0.001
	Prophylactic post-op antibiotics (vs none/missing)	1.69(0.86 to 3.31)	0.126
**Multivariate logistic regression**			
**Type of risk factor**	**Variable**	**OR (95% CI)**	**p-value**
Non-modifiable prior to surgery	BMI (per kg/m²)	1.08(1.03 to 1.14)	0.001
	ASA (per unit)	1.01(0.48 to 2.11)	0.989
	Diabetic requiring insulin (vs not requiring insulin for glycaemic control)	3.34(0.90 to 12.39)	0.071
Modifiable at time of surgery	Chlorhexadine (vs betadine)	0.58(0.30 to 1.134)	0.109
	Drain (vs none)	2.92(1.41 to 6.04)	0.004
	Staples (vs SC suture)	3.13(1.50 to 6.56)	0.002

Sequelae

Median length of post-operative hospital stay was 5 days (Range 1–71, IQR 4-8). In those that developed infection, there was clinical delay in discharge in 18/54 (33.3%). In the 37 patients with SSI who required adjuvant chemotherapy or radiotherapy, 11 (29.7%) experienced delay in commencing therapy directly or indirectly as a result of infection.

For peer review only - http://bmjopen.bmj.com/site/about/guidelines.xhtml

Approximately half of all infections were diagnosed following discharge and 5 (1.5%) patients necessitated readmission to hospital following diagnosis of infection. 30 (55.6%) patients had evidence of skin dehiscence, 14 of which required wound packing. As a result of wound complications; 4 patients returned to theatre, 1 patient was admitted to level 2/3 care and 2 patients required negative pressure wound therapy.

Discussion

Modelled on the highly successful trainee-led research collaboratives in other surgical specialties, the surgical gynaecological research network (SGRN) was formed in 2015 to address the paucity of clinical research in gynaecological cancer surgery. Our study provides a snapshot of current practice of the pre-, intra and post-operative prevention and management of surgical site infection in women undergoing open gynaecological oncology surgery across the UK.

Our study demonstrates that SSI is highly prevalent within the gynaecological oncology population who undergo laparotomy with an incidence of 15.9%; ten times higher than the incidence reported in the NHS Surgical Site Infection Surveillance Programme for women undergoing abdominal hysterectomy in 2015/2016 [9]. The high burden of complications is likely to reflect a combination of patient, surgical and disease-specific factors. Unsurprisingly, infection is prevalent in patients with a high BMI, which perhaps explains the greater incidence observed in those within the endometrial cancer subgroup and in those with diabetes, given shared aetiological factors. Cancer and its treatments can result in immune deficiency making cancer patients in general undergoing surgery a high risk group, but in particular gynaecology oncology patients, with the contaminated nature and complexity of the procedures required [10].

In a small proportion of patients, SSI is severe and/or life threatening, necessitating return to theatre or advanced wound care and prolonged hospital stays. Our study provides evidence that this delays time to delivery of adjuvant cancer treatments.in a significant proportion of patients with SSI.withthe potential to impact upon response to treatment and overall cancer specific survival [10–12]. Additional data, however, is needed to evaluate the impact of these delays on progression and mortality further.

In this study, infection occurred in patients even where compliance with local infection prophylaxis standards were high. Analysis of the micro-organisms cultured from patients affected showed that E-Coli and Enterobacter species predominated. This is in keeping with other studies of wound infection post-hysterectomy[13–15] and the vaginal flora of our predominately post-menopausal cohort[16]. Previous studies have determined that up to 15% of cultured organisms may be resistant to broad spectrum combinations of antibiotics [15]. In our study, sensitivities of the microorganisms cultured from affected patients were not readily available. Further studies would be required to determine whether resistance to current antibiotic regimens could explain the high prevalence of SSI in this population.

For peer review only - http://bmjopen.bmj.com/site/about/guidelines.xhtml

The use of drains and staples for wound closure were positively associated with SSI in our population. These devices are commonly used in patients thought to be at high risk of SSI and therefore, the associations seen in this study may not be causative. Surgical drains are often used to drain body fluids away from a dead space to promote wound healing and prevent potential SSI. Although there is conflicting data as to whether the drains are associated with an increase in SSI, no studies have shown a reduction in SSI following the use of surgical drains[17–20]. Judicious use of drains and timely removal is therefore, indicated. Staples are an efficient way to close a linear incision and may be preferred by some surgeons after particularly long or technically difficult cases. Various randomised control studies and subsequent meta-analyses fail to show that the use of sutures over staples resulted in any difference in SSI in gynaecological surgery [21–25].

At a time when many NHS trusts are keen to invest in enhanced recovery programmes to encourage a speedy and proactive approach to recovery for patients, this audit demonstrates a need for effective management of wounds to minimise the incidence of wound infection and provide effective management rapidly when it does occur. These data support the development of a stratified care bundle to reduce delay to adjuvant treatment associated with SSI focussing on women with high BMI and poorly controlled diabetes.

Strengths and Limitations

To the best of our knowledge, this study is the first to comprehensively review current practice surrounding the prevention and management of SSI in open gynaecological cancer surgery. The UKGOSOC study found a slightly lower wound complication rate of 9.6% across all types of gynaecological cancer surgery in ten UK gynaecological cancer centres [1]. However, this study included patients undergoing both laparoscopic and open surgery. Laparoscopic surgery has previously been shown to have lower incidence of post-operative complications such as wound infection [26–28]. We have therefore chosen to examine care practices in women undergoing open surgery specifically.

The strength of our review of practice is that the data were prospectively collected using standardised data collection forms at 12 participating centres from around the UK. As this was a review of practice, data from all consecutive surgeries were included as patient recruitment was not required. We included all reported cases of SSI (including superficial incisional infections) and their sequelae; thus, not presupposing, that particular classes of SSI would have an impact on patient recovery and subsequent cancer treatment. Despite our use of a standardised proforma and the use of a pre-agreed definition of SSI, there remained a potential for variation in its interpretation from site to site. This may have contributed to the outlier unit with the disproportionately high rate of SSI. However, this type of variation is likely to also reflect that seen in day-to-day practice. Twelve of the 44 units which offer tertiary level gynaecological cancer surgery participated in this review. No units from the South West or Wales participated. The inclusion of these units together with a longer data collection window may have led to a larger sample, allowing the identification of further potential risk factors for SSI and highlighting additional practices that may reduce wound infection rates. The identification of risk factors and good practice points will enable us to develop a tool to personalise patient care plans with the aim of reducing surgical site infection.

For peer review only - http://bmjopen.bmj.com/site/about/guidelines.xhtml

Conclusions

This trainee-led collaborative initiative demonstrates the need to determine whether addressing modifiable risk factors can prevent SSI in gynaecological cancer surgery. Ultimately, a reduction in delayed discharge and subsequent delays in the commencement of adjuvant treatment could result in improved patient outcomes.

References

1 Iyer R, Gentry-Maharaj A, Nordin A, *et al.* Predictors of complications in gynaecological oncological surgery: a prospective multicentre study (UKGOSOC-UK gynaecological oncology surgical outcomes and complications). *Br J Cancer* 2015;**112**:475–84. doi:10.1038/bjc.2014.630

2 NICE Clinical Guidelines. Surgical site infections: prevention and treatment. 2008.

3 Chief Medical Officer and Chief Nursing Officer. Surveillance of Healthcare Associated Infections. 2003.

4 Public Health England. Surveillance of Surgical Site Infections in NHS hospitals in England: 2016-2017. 2017.

5 Dripps RD. New classification of physical status. *Anesthesiology* 1963;**24**:111.

6 Aletti GD, Dowdy SC, Podratz KC, *et al.* Relationship among surgical complexity , short-term morbidity , and overall survival in primary surgery for advanced ovarian cancer. *Obstet Gynecol* 2007;:1–7. doi:10.1016/j.ajog.2007.10.495

7 Center for Disease Control. Chapter 9: Surgical Site Infection (SSI) Event. In: *National Healthcare Safety Network (NHSN) Patient Safety Component Manual*. 2015. https://www.cdc.gov/nhsn/pdfs/pscmanual/9pscssicurrent.pdf

8 Public Health England. Protocol for the Surveillance of Surgical Site Infection. 2013.

9 Public Health England. Surveillance of Surgical Site Infections in NHS hospitals in England. 2016.

10 Tran CW, McGree ME, Weaver AL, *et al.* Surgical site infection after primary surgery for epithelial ovarian cancer: Predictors and impact on survival. *Gynecol Oncol* 2015;**136**:278–84. doi:10.1016/j.ygyno.2014.12.007

11 Haruki K, Shiba H, Fujiwara Y, *et al.* Negative impact of surgical site infection on long-term outcomes after hepatic resection for colorectal liver metastases. *Anticancer Res* 2013;**33**:1697–703.

12 Buettner S, Ethun CG, Poultsides G, *et al.* Surgical Site Infection Is Associated with Tumor Recurrence in Patients with Extrahepatic Biliary Malignancies. *J Gastrointest Surg* 2017;**21**:1813–20. doi:10.1007/s11605-017-3571-2

13 Miranda C, Alados JC, Molina JM, *et al.* Posthysterectomy wound infection. A review. *Diagn Microbiol Infect Dis* 1993;**17**:41–4.http://www.ncbi.nlm.nih.gov/pubmed/8359004 (accessed 14 Oct 2018).

For peer review only - http://bmjopen.bmj.com/site/about/guidelines.xhtml

14 Roy S, Wilkins J, Galaif E, *et al.* Comparative efficacy and safety of cefmetazole or cefoxitin in the prevention of postoperative infection following vaginal and abdominal hysterectomy. *J Antimicrob Chemother* 1989;**23**:109–17. doi:10.1093/jac/23.suppl_D.109

15 Yerushalmy A, Reches A, Lessing JB, *et al.* Characteristics of microorganisms cultured from infected wounds post-hysterectomy. *Eur J Obstet Gynecol Reprod Biol* 2008;**141**:169–72. doi:10.1016/j.ejogrb.2008.07.024

16 Ginkel PD, Soper DE, Bump RC, *et al.* Vaginal Flora in Postmenopausal Women: The Effect of Estrogen Replacement. Wiley-Liss, Inc 1993. https://www.ncbi.nlm.nih.gov/pmc/articles/PMC2364299/pdf/IDOG-01-094.pdf (accessed 14 Oct 2018).

17 Kaya E, Paksoy E, Ozturk E, *et al.* Subcutaneous closed-suction drainage does not affect surgical site infection rate following elective abdominal operations: a prospective randomized clinical trial. *Acta Chir Belg*;**110**:457–62.http://www.ncbi.nlm.nih.gov/pubmed/20919669 (accessed 14 Oct 2018).

18 Barbadoro P, Marmorale C, Recanatini C, *et al.* May the drain be a way in for microbes in surgical infections? *Am J Infect Control* 2016;**44**:283–8. doi:10.1016/j.ajic.2015.10.012

19 Reiffel AJ, Barie PS, Spector JA. A Multi-Disciplinary Review of the Potential Association between Closed-Suction Drains and Surgical Site Infection. *Surg Infect (Larchmt)* 2013;**14**:244–69. doi:10.1089/sur.2011.126

20 Mujagic E, Zeindler J, Coslovsky M, *et al.* The association of surgical drains with surgical site infections – A prospective observational study. *Am J Surg* Published Online First: 19 June 2018. doi:10.1016/j.amjsurg.2018.06.015

21 Hemming K, Pinkney T, Futaba K, *et al.* A systematic review of systematic reviews and panoramic meta-analysis: staples versus sutures for surgical procedures. *PLoS One* 2013;**8**:e75132. doi:10.1371/journal.pone.0075132

22 Mackeen AD, Berghella V, Larsen M-L. Techniques and materials for skin closure in caesarean section. In: Berghella V, ed. *Cochrane Database of Systematic Reviews*. Chichester, UK: : John Wiley & Sons, Ltd 2012. CD003577. doi:10.1002/14651858.CD003577.pub3

23 Tuuli MG, Rampersad RM, Carbone JF, *et al.* Staples Compared With Subcuticular Suture for Skin Closure After Cesarean Delivery. *Obstet Gynecol* 2011;**117**:682–90. doi:10.1097/AOG.0b013e31820ad61e

24 Kuroki LM, Mullen MM, Massad LS, *et al.* Wound Complication Rates After Staples or Suture for Midline Vertical Skin Closure in Obese Women: A Randomized Controlled Trial. *Obstet Gynecol* 2017;**130**:91–9. doi:10.1097/AOG.0000000000002061

For peer review only - http://bmjopen.bmj.com/site/about/guidelines.xhtml

25 Mackeen AD, Schuster M, Berghella V. Suture versus staples for skin closure after cesarean: a metaanalysis. *Am J Obstet Gynecol* 2015;**212**:621.e1-621.e10. doi:10.1016/j.ajog.2014.12.020

26 Mahdi H, Gojayev A, Buechel M, *et al.* Surgical Site Infection in Women Undergoing Surgery for Gynecologic Cancer. *Int J Gynecol Cancer* 2014;**24**:779–86. doi:10.1097/IGC.0000000000000126

27 Walker JL, Piedmonte MR, Spirtos NM, *et al.* Laparoscopy compared with laparotomy for comprehensive surgical staging of uterine cancer: Gynecologic Oncology Group Study LAP2. *J Clin Oncol* 2009;**27**:5331–6. doi:10.1200/JCO.2009.22.3248

28 Park J-Y, Kim D-Y, Kim J-H, *et al.* Laparoscopic versus open radical hysterectomy in patients with stage IB2 and IIA2 cervical cancer. *J Surg Oncol* 2013;**108**:63–9. doi:10.1002/jso.23347

Figure Legends

Figure 1: Funnel plot of SSI by UK cancer centre: Funnel plot of the number of reported surgical site infections for each gynaecological cancer unit versus effective sample size for each unit. The funnels correspond to 95% confidence limits (dashed lines). The outlier unit is denoted by a triangle, whilst units with SSI incidence within the 95% confidence limits are marked with a circle.

For peer review only

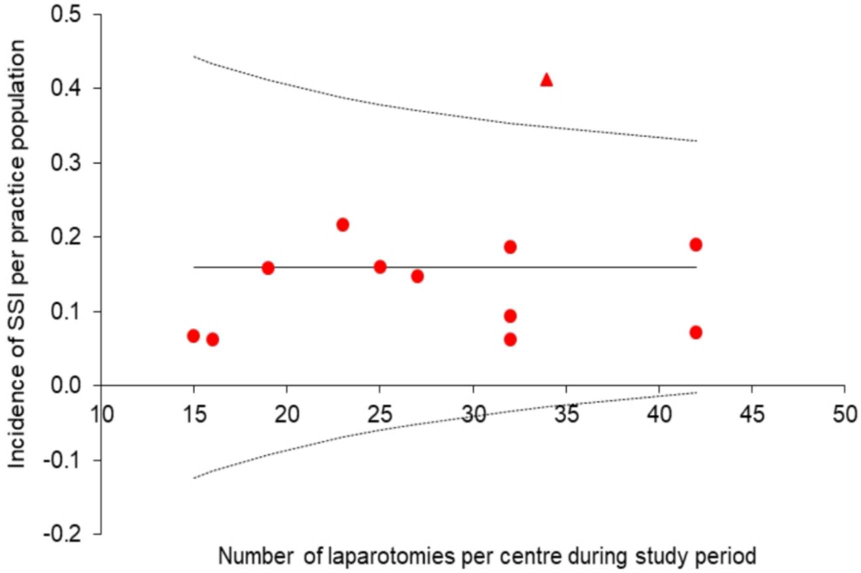

Figure 1: Funnel plot of SSI by UK cancer centre: Funnel plot of the number of reported surgical site infections for each gynaecological cancer unit versus effective sample size for each unit. The funnels correspond to 95% confidence limits (dashed lines). The outlier unit is denoted by a triangle, whilst units with SSI incidence within the 95% confidence limits are marked with a circle.

Supplementary Table A: Modified surgical complexity scoring system based upon complexity and number of surgical procedures performed

Adapted from [6]

Procedure	Points
Total Hysterectomy- Bilateral Salpingo-Oophorectomy (TH-BSO)	1
Omentectomy	1
Pelvic lymphadenectomy	1
Paraaortic lymphadenectomy	1
Pelvic peritoneum stripping	1
Abdominal peritoneum stripping	1
Rectosigmoidectomy EEA	3
Large bowel resection	2
Diaphragm stripping/resection	2
Splenectomy	2
Liver resection	2
Small bowel resection	1
Resection of tail of pancreas	5
Cholecystectomy	5
Partial gastrectomy	5

Complexity score groups

1. Standard/ (low) ≤3
2. Intermediate 4-8
3. Ultra-radical (high) ≥8

For peer review only

Supplementary Table B:

(adapted from [7])

Term	Definition	Criteria (Any 1 of criteria below)
Superficial incisional infection	SSI within 30 days of surgery involving the skin or subcutaneous tissue of the incision	1. Purulent drainage from incision<br>2. Positive microbiological culture/pus cells present on swab from incision<br>3. Two of:<br>a. pain or tenderness<br>b. swelling<br>c. redness<br>4. the superficial incision is deliberately opened by a surgeon to manage the infection<br>5. clinician diagnoses a superficial incisional infection.
Deep incisional infection:	SSI involving the deep tissues (i.e. fascial and muscle layers) that occurs within 30 days of surgery<br>.	1. Purulent drainage from the deep incision<br>2. Positive microbiological culture/pus cells present on swab from fluid from deep incision<br>3. Spontaneous dehiscence/opening of wound by surgeon AND patient has at least 1 of:<br>a. fever (>38°C)<br>b. localized pain or tenderness<br>4. Radiologically, histologically or surgically diagnosed abscess involving the deep incision.<br>5. Clinician diagnoses a deep incisional SSI
Deep organ space infection	SSI involving any part of the body deeper than the fascial and muscle layers that was opened or manipulated during the operative procedure occurring within 30 days of the procedure	Patient has at least two of the following: fever (>38.0°C), hypotension, nausea, vomiting, abdominal pain or tenderness AND has either organisms identified from tissue or fluid or positive blood cultures and radiological evidence of abscess or infection
Patient reported SSI	SSI reported by patient to have occurred within 30 days of surgery	1. Discharge pus AND antibiotics prescribed<br>2. Clinical signs* AND dehiscence<br>3. Clinical signs* AND antibiotics prescribed<br><br>*clinical signs – at least 2 of the following must be present: pain, heat, redness or swelling

For peer review only

For peer review only

Revised Standards for Quality Improvement Reporting Excellence (SQUIRE 2.0)

September 15, 2015

Text Section and Item Name	Section or Item Description	
Notes to authors	The SQUIRE guidelines provide a framework for reporting new knowledge about how to improve healthcare<br>The SQUIRE guidelines are intended for reports that describe system level work to improve the quality, safety, and value of healthcare, and used methods to establish that observed outcomes were due to the intervention(s).<br>A range of approaches exists for improving healthcare. SQUIRE may be adapted for reporting any of these.<br>Authors should consider every SQUIRE item, but it may be inappropriate or unnecessary to include every SQUIRE element in a particular manuscript.<br>The SQUIRE Glossary contains definitions of many of the key words in SQUIRE.<br>The Explanation and Elaboration document provides specific examples of well-written SQUIRE items, and an in-depth explanation of each item.<br>Please cite SQUIRE when it is used to write a manuscript.	As you review the manuscript, place a checkmark in this column for each SQUIRE item that is appropriately addressed in the manuscript. Remember that not every item is necessary in every manuscript.
Title and Abstract		
1. Title	Indicate that the manuscript concerns an initiative to improve healthcare (broadly defined to include the quality, safety, effectiveness, patient-centeredness, timeliness, cost, efficiency, and equity of healthcare)	***Yes, inclusion in the title of audit and service evaluation i.e. methods through***

For peer review only

		which healthcare is improved.
2. Abstract	a. Provide adequate information to aid in searching and indexing<br>b. Summarize all key information from various sections of the text using the abstract format of the intended publication or a structured summary such as: background, local problem, methods, interventions, results, conclusions	***a. Keywords for indexing included: gynaecological oncology, wound management, surgical site infection, medical education,***<br>***b. Structured abstract as per BMJ Open guidance***
Introduction	*Why did you start?*	
3. Problem Description	Nature and significance of the local problem	***Introduction***
4. Available knowledge	Summary of what is currently known about the problem, including relevant previous studies	***Introduction***
5. Rationale	Informal or formal frameworks, models, concepts, and/or theories used to explain the problem, any reasons or assumptions that were used to develop the intervention(s), and reasons why the intervention(s) was expected to work	***Introduction***
6. Specific aims	Purpose of the project and of this report	***Introduction***
Methods	*What did you do?*	
7. Context	Contextual elements considered important at the outset of introducing the intervention(s)	***Introduction***
8. Intervention(s)	Description of the intervention(s) in sufficient detail that others could reproduce it<br>Specifics of the team involved in the work	***Methods***<br>***Contributions,***
9. Study of the Intervention(s)	Approach chosen for assessing the impact of the intervention(s)<br>Approach used to establish whether the observed outcomes were due to the intervention(s)	***Methods***<br>***n/a***

For peer review only

10. Measures	Measures chosen for studying processes and outcomes of the intervention(s), including rationale for choosing them, their operational definitions, and their validity and reliability<br>Description of the approach to the ongoing assessment of contextual elements that contributed to the success, failure, efficiency, and cost<br>Methods employed for assessing completeness and accuracy of data	***Methods***<br>***n/a***<br>***Methods***
11. Analysis	Qualitative and quantitative methods used to draw inferences from the data<br>Methods for understanding variation within the data, including the effects of time as a variable	***Statistical analysis***
12. Ethical Considerations	Ethical aspects of implementing and studying the intervention(s) and how they were addressed, including, but not limited to, formal ethics review and potential conflict(s) of interest	***Methods***
Results	*What did you find?*	
13. Results	Initial steps of the intervention(s) and their evolution over time (*e.g.*, time-line diagram, flow chart, or table), including modifications made to the intervention during the project<br>Details of the process measures and outcome<br>Contextual elements that interacted with the intervention(s)<br>Observed associations between outcomes, interventions, and relevant contextual elements<br>Unintended consequences such as unexpected benefits, problems, failures, or costs associated with the intervention(s).<br>Details about missing data	***Data collection***<br>***Results section***<br>***Table 3***<br>***Table 1 & 2***<br>***Table 4***<br>***n/a***<br>***Data collection***
Discussion	*What does it mean?*	
14. Summary	Key findings, including relevance to the rationale and specific aims<br>Particular strengths of the project	***Yes, see main discussion***
15. Interpretation	Nature of the association between the intervention(s) and the outcomes<br>Comparison of results with findings from other publications<br>Impact of the project on people and systems<br>Reasons for any differences between observed and anticipated outcomes, including the influence of context<br>Costs and strategic trade-offs, including opportunity costs	***Yes, see main discussion***<br>***n/a***

For peer review only

16. Limitations	Limits to the generalizability of the work<br>Factors that might have limited internal validity such as confounding, bias, or imprecision in the design, methods, measurement, or analysis<br>Efforts made to minimize and adjust for limitations	***Yes, see strengths and limitations section***
17. Conclusions	Usefulness of the work<br>Sustainability<br>Potential for spread to other contexts<br>Implications for practice and for further study in the field<br>Suggested next steps	***Yes, see main discussion and conclusion***
Other information		
18. Funding	Sources of funding that supported this work. Role, if any, of the funding organization in the design, implementation, interpretation, and reporting	***Yes, see funding statement***

For peer review only
